# Molecular characterization and phylogenetic relatedness of dog-derived Rabies Viruses circulating in Cameroon between 2010 and 2016

**DOI:** 10.1371/journal.pntd.0006041

**Published:** 2017-10-30

**Authors:** Serge Alain Sadeuh-Mba, Jean Blaise Momo, Laura Besong, Sévérin Loul, Richard Njouom

**Affiliations:** 1 Virology Service, Centre Pasteur du Cameroun, Yaounde, Centre region, Cameroon; 2 Ministry of Livestock, Fisheries and Animal Industries (MINEPIA), Yaounde, Centre region, Cameroon; Wistar Institute, UNITED STATES

## Abstract

Rabies is enzootic among dog populations in some parts of Cameroon and the risk of human rabies is thought to be steadily high in these regions. However, the molecular epidemiology of circulating Rabies Virus (RABV) has been hardly considered in Cameroon as well as in most neighboring central African countries. To address this fundamental gap, 76 nucleoprotein (N) gene sequences of dog-derived RABV were obtained from 100 brain specimens sampled in Cameroon from 2010 to 2016. Studied sequences were subjected to molecular and phylogenetic analyses with reference strains retrieved from databases. The 71 studied Africa-1 isolates displayed 93.5–100% nucleotide (nt) and 98.3–100% amino-acid (aa) identities to each other while, the 5 studied Africa-2 isolates shared 99.4–99.7% sequence similarities at nt and aa levels. Maximum Likelihood based phylogenies inferred from nucleotide sequences confirmed all studied RABV isolates as members of the dog-related species 1 of the *Lyssavirus* genus. Individual isolates could be unambiguously assigned as either the Africa-1 subclade of the Cosmopolitan clade or the Africa 2 clade. The Africa-1 subclade appeared to be more prevalent and diversified. Indeed, 70 studied isolates segregated into 3 distinct circulating variants within Africa-1a lineage while a unique isolate was strikingly related to the Africa-1b lineage known to be prevalent in the neighboring Central African Republic and eastern Africa. Interestingly, all five Africa-2 isolates fell into the group-E lineage even though they appeared to be loosely related to databases available reference RABV; including those previously documented in Cameroon. This study uncovered the co-circulation of several Africa-1 and Africa-2 lineages in the southern regions of Cameroon. Striking phylogenetic outcasts to the geographic differentiation of RABV variants indicated that importation from close regions or neighboring countries apparently contributes to the sustainment of the enzootic cycle of domestic rabies in Cameroon.

## Author summary

Rabies has been repeatedly reported among dog populations in Cameroon, especially in Yaounde, its capital city. However, the relative rates and genetic variability of Rabies Virus (RABV) variants circulating among dog populations in Cameroon are still to be documented. This study aimed to estimate the frequency and genetic diversity of RABV isolates originating from rabid dogs in the southern regions of Cameroon from 2010 to 2016. Overall, 76 of the 100 dog-derived RABV isolates sampled in Cameroon from 2010 to 2016 were successfully characterized. Our findings revealed that studied isolates belonged to the dog-related species 1 of the *Lyssavirus genus*, specifically 70 Africa-1a, 1 Africa-1b and 5 Africa-2 group-E lineages. The general phylogenetic pattern suggested an in-country geographic differentiation of the circulating RABV variants. This apparent geographic differentiation was contradicted by striking outcasts indicating importation from close or distant regions. Overall, this study uncovered the co-circulation of several Africa-1 and Africa-2 lineages in some southern regions of Cameroon, thus providing base-line molecular data that would be of interest for future stages of implementation of the rabies surveillance and control plan that is being setup in Cameroon.

## Introduction

Rabies is a neglected lethal neurological disease which has a case-fatality rate of almost 100% [1–3]. It causes an estimated 59,000 human deaths primarily in developing and low-income countries where the disease is endemic in animal populations [1,3]. Bites from rabid domestic dogs account for over 99% of the human rabies cases [3,4], most of which occur in Asia and Africa [5,6]. Post-exposure prophylaxis (PEP) efficiently prevents disease development in humans bitten by rabid animals when administrated immediately. Unfortunately, PEP is often unavailable in all settings or not affordable in many developing countries [3].

Canine rabies has been shown to be endemic in Cameroon and relatively higher frequencies of rabid dogs have been reported in urban settings compared to rural areas [7,8]. In the absence of a multiannual active national surveillance and control strategy, the actual burden of human and animal rabies in Cameroon is likely underestimated as in other African countries [3,9–12]. Some rabies control interventions, such as yearly discount of pet vaccination and irregular radio communication campaigns, are conducted in Cameroon but their actual impact remains unknown [8,9,13].

The etiological agent of rabies, the Rabies Virus (RABV), of the genus *Lyssavirus and* family *Rhabdoviridae*, has a single-stranded RNA genome of approximately 12 kb in length and of negative polarity [14]. The RABV genome consists of five genes encoding the nucleoprotein (N), the phosphoprotein (P), the matrix protein (M), the glycoprotein (G) and the large protein which is the polymerase (L). These five genes N, P, M, G, and L are separated by intergenic regions of variable lengths [14,15]. Like other RNA viruses, RABV displays high rates of mutation due to the lack of proofreading activity of the L protein [16]. RABV is the only virus among the 16 known *Lyssavirus* species found worldwide in a wide range of mammalian reservoirs of the orders *Chiroptera* and *Carnivora* [17–20]. It has been recently demonstrated that individual gene or complete genome sequences of RABV isolates segregate into two major phylogenetic clusters gathering bat- and dog-derived RABV isolates respectively. Within these clades, isolates fall into several major clades [21].

Bat-derived RABV isolates have been shown to circulate specifically in the New World mainly among bats and, to a lesser extent, in some terrestrial carnivores such as skunks (*Mephitis mephitis*) and raccoons (*Procyon lotor*) [22–25]. Conversely, dog-specific RABV isolates have been documented worldwide mainly among domestic dogs, but also among wild-living carnivores comprising foxes and raccoon dogs in Europe [26], foxes in the Middle East [27], raccoon dogs and ferret-badgers in Asia [28–30], skunks, foxes, coyotes and mongooses in the Americas [23,24], African civet and mongooses in Africa [31,32]. Within the divergent dog-specific cluster, six major well-defined clades, respectively assigned as Africa-2, Africa-3, Arctic-related, Asian, Cosmopolitan and Indian clades, have been documented [21,33].

In particular, molecular studies in Africa uncovered the presence of Cosmopolitan, Africa-2, and Africa-3 clades. All these clades comprise classical RABV species that segregate into several subclades and lineages varying by geographic area, virus variability, and reservoir species in Africa [21,33–37]. Two major subclades are defined by field RABV isolates of the Cosmopolitan clade: Africa-1 and Africa-4. Africa-4 has been recently identified in northern Africa [21,34] while Africa-1 subclade has been shown to circulate in the northern, eastern and southern parts of Africa [33,38]. Africa-1a lineage has been suggested to have a very broad distribution across Africa. It is predominant in northern and eastern Africa [38–40] and has also been previously reported in Cameroon, Gabon, Equatorial Guinea, Ghana and Madagascar [38,41,42]. Africa-1b lineage circulates mainly in eastern and southern Africa [38,39,43,44].

Africa-2 lineages are uninterruptedly found across West and Central Africa [35–37,42] and has been shown to co-circulate with the Africa-1 lineages in Nigeria and Central African Republic [36,38,42,44–46]. Although Africa-1 and Africa-2 lineages have been documented in several domestic and wild carnivore species, domestic dogs are virtually the only population essential for maintaining canid variants in some parts of Africa [47]. Conversely, wild canids have been suggested to contribute to the sustainment of canine rabies cycles in specific geographic locations in South Africa, Namibia and Zimbabwe [32,48–50].

The third Africa-3 clade is well adapted to mongooses [31,51,52] and is sustained through an independent epidemiological cycle (distinct from that of dog RABV) within viverrid species in southern Africa [31,32,51,53].

Although RABV has been continuously reported in Cameroon [7,8,54], its molecular epidemiology among dog populations have not yet been documented. Less than five genomic sequences of dog-related Africa-1 and Africa-2 RABV originating from Cameroon have been described in previous studies [21,36,38,44]. Based on very limited sequence data available so far in Cameroon, it was thought that there may be an in-country geographic differentiation of dog-derived RABV; with Africa-1 isolates found in the Center region including the capital city, Yaounde, and Africa-2 isolates detected in the northern part of Cameroon.

The purpose of this study was to provide insight into the frequency, genetic variability and phylogenetic relatedness of the RABV isolates derived from rabid dog in Cameroon. Interestingly, this study uncovered the co-circulation of both Africa-1 and Africa-2 in two southern regions of Cameroon and indicated that they circulate in close proximity between neighboring administrative regions of Cameroon as well as between Cameroon and neighboring countries.

## Results

### Geographic and temporal distribution of the RABV-positive specimens analyzed

**Fig 1:**
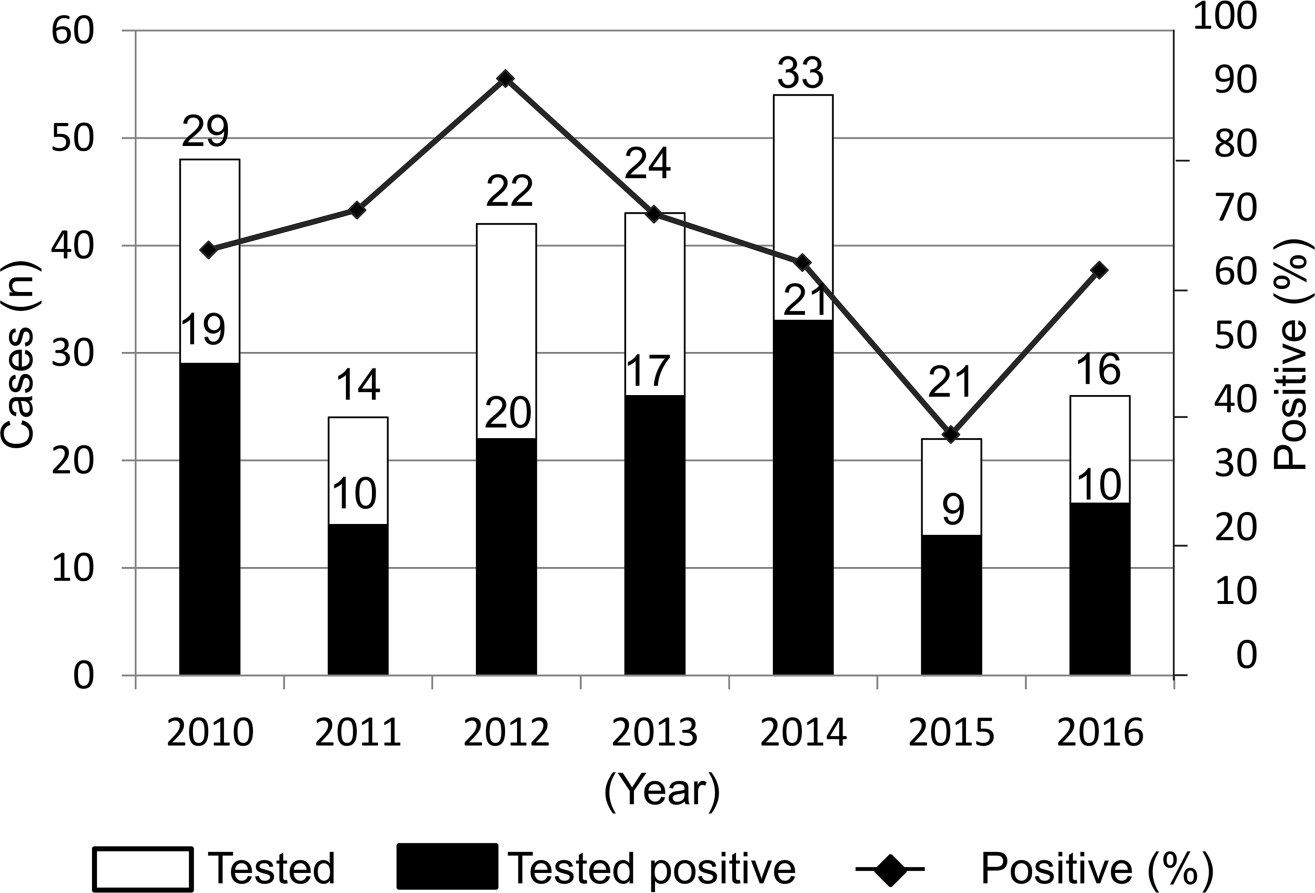
Annual distribution of laboratory confirmed rabies cases among dogs originating from the southern regions of Cameroon, 2010–2016.

From January 2010 to December 2016, a total of 163 animal specimens were analyzed for rabies diagnosis at CPC, comprising 159 specimens originating from domestic dogs and 4 specimens from other animal species (1 cat, 1 cow, 1 monkey and 1 pig). Overall, 65.4% (104/159) of all dog specimens analyzed were from the Center region amongst which 61.5% (64/104) originated from the capital city, Yaounde where CPC is located. All specimens from cat, cow, monkey and pig were found rabies-negative whereas 66.0% (105/159) of dog specimens were confirmed rabies-positive. The number of dog specimens analyzed from 2010 to 2016 as well as annual rates of positive samples were variable as depicted in Fig 1. Overall, 100 of the 105 rabies positive specimens were available for molecular characterization in this study. They originated from the Center region (68 samples amongst which 46 from Yaounde), East region (1 sample), Littoral region (3 samples), North West region (8 samples), West region (14 samples), South West region (3 samples) and South region (3 samples) (Table 1).

**Table 1:** Summary of the characteristics and genotyping results of the Rabies Virus isolates derived from brain specimens of rabid dogs enrolled in this study.

Virus Name	Origin^a^			Host species	Source	Phylogenetic clade (subclade)^b^	Sequence Accession number
		District codes	Regions of origin					Districts of origin	
14V-9183	CEN-BOK	Centre	Bokito	Dog (Canis familiaris)	Original brain	Cosmopolitan (AF1a)	MF537529
13V-1970	CEN-EKO	Centre	Ekoumtik	Dog (Canis familiaris)	Original brain	Cosmopolitan (AF1a)	MF537550
14V-1505	CEN-MAT	Centre	Matomb	Dog (Canis familiaris)	Original brain	Not applicable	/
14V-1507	CEN-MAT	Centre	Matomb	Dog (Canis familiaris)	Original brain	Not applicable	/
14V-2281	CEN-MBA	Centre	Mbalmayo	Dog (Canis familiaris)	Original brain	Cosmopolitan (AF1a)	MF537549
14V-6062	CEN-MBA	Centre	Mbalmayo	Dog (Canis familiaris)	Original brain	Cosmopolitan (AF1a)	MF537554
14V-7214	CEN-MBA	Centre	Mbalmayo	Dog (Canis familiaris)	Original brain	Cosmopolitan (AF1a)	MF537551
11V-5200	CEN-MON	Centre	Monatélé	Dog (Canis familiaris)	Original brain	Cosmopolitan (AF1a)	MF537541
11V-6266	CEN-MON	Centre	Monatélé	Dog (Canis familiaris)	Original brain	Cosmopolitan (AF1a)	MF537536
11V-18499	CEN-NTU	Centre	Ntui	Dog (Canis familiaris)	Original brain	Cosmopolitan (AF1a)	MF537542
11V-19185	CEN-NTU	Centre	Ntui	Dog (Canis familiaris)	Original brain	Not applicable	/
12V-2731	CEN-NTU	Centre	Ntui	Dog (Canis familiaris)	Original brain	Not applicable	/
16V-608	CEN-NTU	Centre	Ntui	Dog (Canis familiaris)	Original brain	Cosmopolitan (AF1a)	MF537519
10V-5902	CEN-OBA	Centre	Obala	Dog (Canis familiaris)	Original brain	Cosmopolitan (AF1a)	MF537511
11V-18800	CEN-OBA	Centre	Obala	Dog (Canis familiaris)	Original brain	Cosmopolitan (AF1a)	MF537520
11V-7563	CEN-OBA	Centre	Obala	Dog (Canis familiaris)	Original brain	Cosmopolitan (AF1a)	MF537509
12V-746	CEN-OBA	Centre	Obala	Dog (Canis familiaris)	Original brain	Cosmopolitan (AF1a)	MF537539
13V-5439	CEN-OBA	Centre	Obala	Dog (Canis familiaris)	Original brain	Not applicable	/
14V-1546	CEN-OKO	Centre	Okola	Dog (Canis familiaris)	Original brain	Cosmopolitan (AF1a)	MF537544
16V-2918	CEN-OKO	Centre	Okola	Dog (Canis familiaris)	Original brain	Not applicable	/
12V-1395	CEN-SAA	Centre	Sa'a	Dog (Canis familiaris)	Original brain	Cosmopolitan (AF1a)	MF537558
16V-1728		CEN-SAA	Centre	Sa'a		Dog (Canis familiaris)	Original brain	Cosmopolitan (AF1a)	MF537547
10V-2182	CEN-YAO	Centre	Yaounde	Dog (Canis familiaris)	Original brain	Cosmopolitan (AF1a)	MF537538
10V-2240	CEN-YAO	Centre	Yaounde	Dog (Canis familiaris)	Original brain	Cosmopolitan (AF1a)	MF537553
10V-2374	CEN-YAO	Centre	Yaounde	Dog (Canis familiaris)	Original brain	Cosmopolitan (AF1a)	MF537543
10V-2510	CEN-YAO	Centre	Yaounde	Dog (Canis familiaris)	Original brain	Cosmopolitan (AF1a)	MF537540
10V-2713	CEN-YAO	Centre	Yaounde	Dog (Canis familiaris)	Original brain	Cosmopolitan (AF1a)	MF537534
10V-3801	CEN-YAO	Centre	Yaounde	Dog (Canis familiaris)	Original brain	Cosmopolitan (AF1a)	MF537537
11V-1662	CEN-YAO	Centre	Yaounde	Dog (Canis familiaris)	Original brain	Cosmopolitan (AF1a)	MF537513
11V-267	CEN-YAO	Centre	Yaounde	Dog (Canis familiaris)	Original brain	Not applicable	/
12V-1202	CEN-YAO	Centre	Yaounde	Dog (Canis familiaris)	Original brain	Cosmopolitan (AF1a)	MF537535
12V-1379	CEN-YAO	Centre	Yaounde	Dog (Canis familiaris)	Original brain	Cosmopolitan (AF1a)	MF537559
12V-3708	CEN-YAO	Centre	Yaounde	Dog (Canis familiaris)	Original brain	Cosmopolitan (AF1a)	MF537548
12V-3857	CEN-YAO	Centre	Yaounde	Dog (Canis familiaris)	Original brain	Cosmopolitan (AF1a)	MF537532
12V-3955	CEN-YAO	Centre	Yaounde	Dog (Canis familiaris)	Original brain	Cosmopolitan (AF1a)	MF537552
12V-4917	CEN-YAO	Centre	Yaounde	Dog (Canis familiaris)	Original brain	Not applicable	/
12V-5365	CEN-YAO	Centre	Yaounde	Dog (Canis familiaris)	Original brain	Cosmopolitan (AF1a)	MF537524
12V-5642	CEN-YAO	Centre	Yaounde	Dog (Canis familiaris)	Original brain	Cosmopolitan (AF1a)	MF537507
12V-5644	CEN-YAO	Centre	Yaounde	Dog (Canis familiaris)	Original brain	Cosmopolitan (AF1a)	MF537531
12V-5932	CEN-YAO	Centre	Yaounde	Dog (Canis familiaris)	Original brain	Cosmopolitan (AF1a)	MF537557
12V-6225	CEN-YAO	Centre	Yaounde	Dog (Canis familiaris)	Original brain	Cosmopolitan (AF1a)	MF537512
12V-6270	CEN-YAO	Centre	Yaounde	Dog (Canis familiaris)	Original brain	Cosmopolitan (AF1a)	MF537518
12V-6272	CEN-YAO	Centre	Yaounde	Dog (Canis familiaris)	Original brain	Not applicable	/
12V-6274	CEN-YAO	Centre	Yaounde	Dog (Canis familiaris)	Original brain	Cosmopolitan (AF1a)	MF537510
12V-805	CEN-YAO	Centre	Yaounde	Dog (Canis familiaris)	Original brain	Not applicable	/
13V-0091	CEN-YAO	Centre	Yaounde	Dog (Canis familiaris)	Original brain	Cosmopolitan (AF1a)	MF537514
13V-1784	CEN-YAO	Centre	Yaounde	Dog (Canis familiaris)	Original brain	Not applicable	/
13V-2178	CEN-YAO	Centre	Yaounde	Dog (Canis familiaris)	Original brain	Cosmopolitan (AF1a)	MF537505
13V-3676	CEN-YAO	Centre	Yaounde	Dog (Canis familiaris)	Original brain	Cosmopolitan (AF1a)	MF537528
13V-4531	CEN-YAO	Centre	Yaounde	Dog (Canis familiaris)	Original brain	Cosmopolitan (AF1a)	MF537533
13V-4610	CEN-YAO	Centre	Yaounde	Dog (Canis familiaris)	Original brain	Cosmopolitan (AF1a)	MF537556
13V-5495	CEN-YAO	Centre	Yaounde	Dog (Canis familiaris)	Original brain	Cosmopolitan (AF1a)	MF537517
13V-6289	CEN-YAO	Centre	Yaounde	Dog (Canis familiaris)	Original brain	Cosmopolitan (AF1a)	MF537515
13V-7152	CEN-YAO	Centre	Yaounde	Dog (Canis familiaris)	Original brain	Cosmopolitan (AF1a)	MF537516
13V-7292	CEN-YAO	Centre	Yaounde	Dog (Canis familiaris)	Original brain	Cosmopolitan (AF1a)	MF537508
13V-7409	CEN-YAO	Centre	Yaounde	Dog (Canis familiaris)	Original brain	Cosmopolitan (AF1a)	MF537525
14V-278	CEN-YAO	Centre	Yaounde	Dog (Canis familiaris)	Original brain	Not applicable	/
14V-4391	CEN-YAO	Centre	Yaounde	Dog (Canis familiaris)	Original brain	Not applicable	/
14V-4642	CEN-YAO	Centre	Yaounde	Dog (Canis familiaris)	Original brain	Cosmopolitan (AF1a)	MF537521
14V-5015	CEN-YAO	Centre	Yaounde	Dog (Canis familiaris)	Original brain	Cosmopolitan (AF1a)	MF537523
14V-5583	CEN-YAO	Centre	Yaounde	Dog (Canis familiaris)	Original brain	Not applicable	/
15V-3509	CEN-YAO	Centre	Yaounde	Dog (Canis familiaris)	Original brain	Not applicable	/
15V-3966	CEN-YAO	Centre	Yaounde	Dog (Canis familiaris)	Original brain	Cosmopolitan (AF1a)	MF537527
15V-5099	CEN-YAO	Centre	Yaounde	Dog (Canis familiaris)	Original brain	Cosmopolitan (AF1a)	MF537526
16V-1501	CEN-YAO	Centre	Yaounde	Dog (Canis familiaris)	Original brain	Not applicable	/
16V-2297	CEN-YAO	Centre	Yaounde	Dog (Canis familiaris)	Original brain	Cosmopolitan (AF1a)	MF537522
16V-470	CEN-YAO	Centre	Yaounde	Dog (Canis familiaris)	Original brain	Cosmopolitan (AF1a)	MF537546
16V-5173		CEN-YAO	Centre	Yaounde		Dog (Canis familiaris)	Original brain	Cosmopolitan (AF1a)	MF537545
14V-4292	EAS-GAR	East	Garoua Boulai	Dog (Canis familiaris)	Original brain	Cosmopolitan (AF1b)	MF537575
15V-1406	LIT-DIB	Littoral	Dibombari	Dog (Canis familiaris)	Original brain	Not applicable	/
14V-4199	LIT-DOU	Littoral	Douala	Dog (Canis familiaris)	Original brain	Cosmopolitan (AF1a)	MF537555
14V-6269		LIT-DOU	Littoral	Douala		Dog (Canis familiaris)	Original brain	Not applicable	/
14V-7840	NOW-BAL	North West	Balikumbat	Dog (Canis familiaris)	Original brain	Cosmopolitan (AF1a)	MF537568
12V-4015	NOW-BAM	North West	Bamenda	Dog (Canis familiaris)	Original brain	Africa-2 (group-E)	MF537580
**13V-6144**	** **	NOW-FUN	North West	Fundong	Dog (Canis familiaris)	Original brain	Africa-2 (group-E)	MF537579
10V-3477	NOW-KUM	North West	Kumbo	Dog (Canis familiaris)	Original brain	Cosmopolitan (AF1a)	MF537570
10V-3478	NOW-KUM	North West	Kumbo	Dog (Canis familiaris)	Original brain	Africa-2 (group-E)	MF537577
14V-1960	NOW-KUM	North West	Kumbo	Dog (Canis familiaris)	Original brain	Africa-2 (group-E)	MF537576
13V-5717	NOW-MBE	North West	Mbengui	Dog (Canis familiaris)	Original brain	Not applicable	/
15V-2175		NOW-BAT	North West	Batibo		Dog (Canis familiaris)	Original brain	Cosmopolitan (AF1a)	MF537560
16V-6323	SOU-AMB	South	AMBAM	Dog (Canis familiaris)	Original brain	Not applicable	/
14V-3979	SOU-EBO	South	Ebolowa	Dog (Canis familiaris)	Original brain	Not applicable	/
15V-6614	SOU-EBO	South	Ebolowa	Dog (Canis familiaris)	Original brain	Not applicable	/
14V-6473	SOW-FON	South West	Fontem	Dog (Canis familiaris)	Original brain	Not applicable	/
14V-6432	SOW-KUM	South West	Kumba	Dog (Canis familiaris)	Original brain	Africa-2 (group-E)	MF537578
14V-6475		SOW-KUM	South West	Kumba		Dog (Canis familiaris)	Original brain	Cosmopolitan (AF1a)	MF537566
12V-007	WES-BAF	West	Bafoussam	Dog (Canis familiaris)	Original brain	Cosmopolitan (AF1a)	MF537506
14V-1636	WES-BAF	West	Bafoussam	Dog (Canis familiaris)	Original brain	Cosmopolitan (AF1a)	MF537565
14V-4880	WES-BAF	West	Bafoussam	Dog (Canis familiaris)	Original brain	Cosmopolitan (AF1a)	MF537567
13V-4215	WES-BAT	West	Batié	Dog (Canis familiaris)	Original brain	Cosmopolitan (AF1a)	MF537530
10V-4586	WES-DSC	West	Dschang	Dog (Canis familiaris)	Original brain	Cosmopolitan (AF1a)	MF537564
11V-18506	WES-DSC	West	Dschang	Dog (Canis familiaris)	Original brain	Cosmopolitan (AF1a)	MF537563
13V-5885	WES-KEK	West	Kekem	Dog (Canis familiaris)	Original brain	Cosmopolitan (AF1a)	MF537561
14V-8641	WES-KEK	West	Kekem	Dog (Canis familiaris)	Original brain	Cosmopolitan (AF1a)	MF537562
10V-2375	WES-PEN	West	Penka Michel	Dog (Canis familiaris)	Original brain	Cosmopolitan (AF1a)	MF537573
10V-2444	WES-PEN	West	Penka Michel	Dog (Canis familiaris)	Original brain	Cosmopolitan (AF1a)	MF537571
10V-3476	WES-PEN	West	Penka Michel	Dog (Canis familiaris)	Original brain	Cosmopolitan (AF1a)	MF537574
10V-3680	WES-PEN	West	Penka Michel	Dog (Canis familiaris)	Original brain	Cosmopolitan (AF1a)	MF537569
10V-3834	WES-PEN	West	Penka Michel	Dog (Canis familiaris)	Original brain	Cosmopolitan (AF1a)	MF537572
13V-0472		WES-SAN	West	Santchou		Dog (Canis familiaris)	Original brain	Not applicable	/

^**a**^ District codes were derived from the respective regions and districts’ names.

^**b**^ Rabies Virus molecular typing from 24 specimens was not applicable because of failure to efficiently amplify the nucleocapsid coding gene (n = 17) or because of unexploitable sequencing results (n = 7).

### Partial sequencing and genetic features of the studied RABV isolates

Of the 100 rabies-positive cases whose brains specimens were analyzed, 76 were confirmed by the molecular analyses performed in this study. The remaining 24 cases without molecular confirmation included 7 cases whose sequences were unexploitable (because of chromatograms with superimposed peaks and/or short reads), 4 cases which showed very weak amplification signals (insufficient for sequencing), and 13 cases which did not amplify. Comparison of the newly determined sequences with homologous sequences obtained from databases identified all 76 RABV isolates as strains of the *Lyssavirus* species 1 and specifically as Africa-1 or Africa-2 lineages. While 71 isolates were identified as belonging to the Africa-1 lineage displaying 93.5–100% nt and 98.3–100% aa identities to each other, 5 RABVs were closely related to Africa-2 isolates sharing 99.4–99.7% sequence similarities at nt and aa levels. Comparison between the studied and few database available Africa-1 RABV sequences from Cameroon showed 93.3–95.2% nt and 93.6–95.4% aa identities. The same analysis showed less sequence divergence between the studied and reference Africa-2 isolates from Cameroon: 96.4–99.0% nt and 94.5–99.7% aa sequence identities.

**Fig 2:**
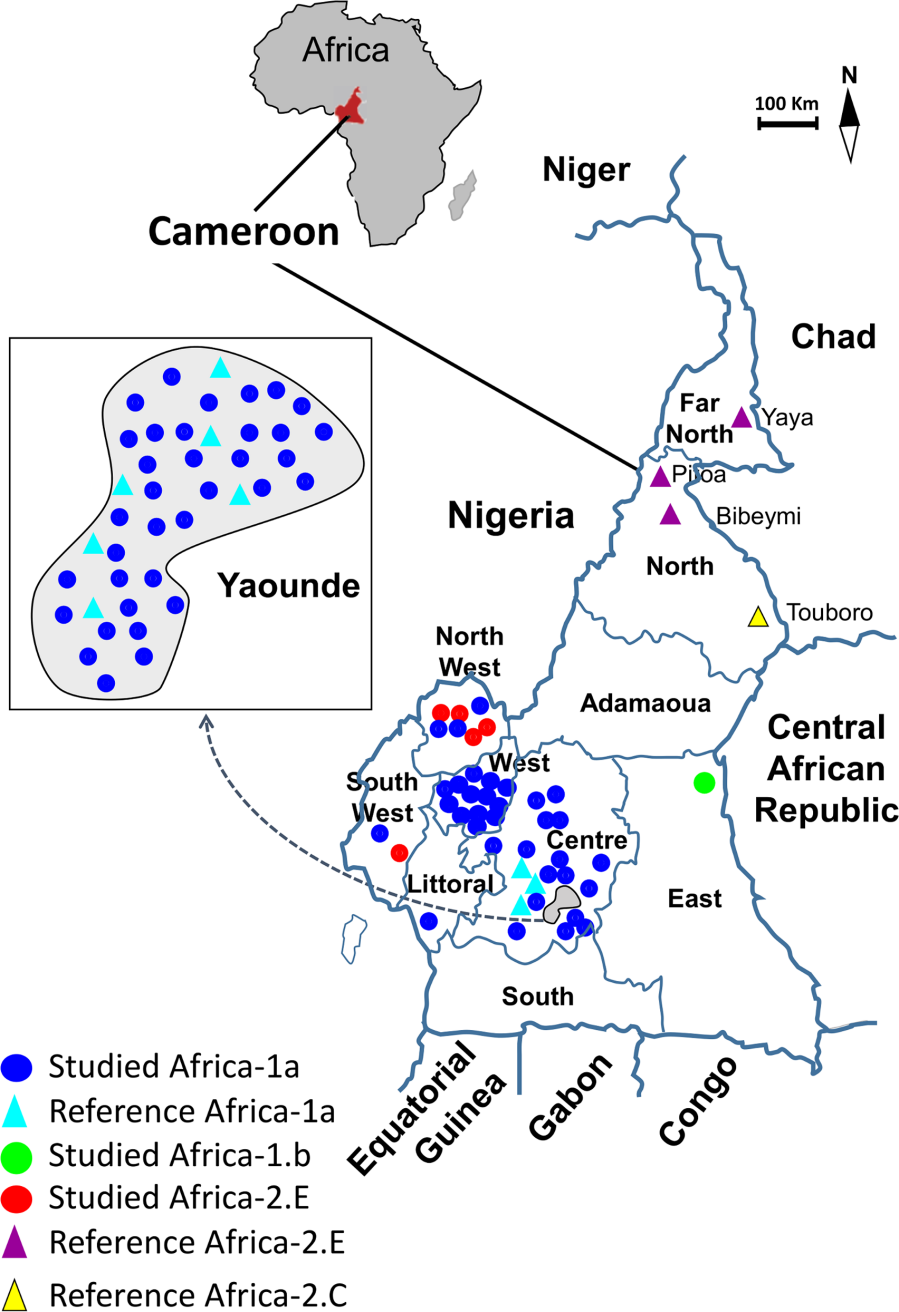
Geographic distribution of the major genetic lineages and variants of the Rabies Virus circulating in Cameroon. Studied isolates are represented by circles color-coded according to their genetic variants: Africa-1a (blue), Africa-1b (green) and Africa-2 group E (red). Strains previously described in Cameroon are also represented and highlighted on the map, with triangles color-coded according to their genetic variants: Reference Africa-1a (blue), Africa-2 group-C (yellow) and Africa-2 group E (purple). The capital city, Yaounde, has been oversized in order to allow better visibility.

Africa-1 isolates, representing 71 of the 76 sequences obtained, were more prevalent and distributed across the southern regions of Cameroon (5 out of the 7 southern regions) (**Fig 2**). In contrast, Africa-2 isolates, detected from 2010 to 2014, were geographically restricted to the two neighboring South West and North West regions (**Fig 2 and Table 1**). This indicates that Africa-1 and Africa-2 RABV co-circulate in some regions of the southern part of Cameroon.

### Phylogenetic relatedness of circulating RABV strains

We analyzed the phylogenetic relationships of the nucleoprotein gene sequences (1040 nt) of the studied RABV isolates with database available homologous sequences representing RABV lineages originating from a wide geographic range in Africa (S1 Table). The resulting Maximum Likelihood phylograms confirmed all newly sequenced RABV isolates from Cameroon as dog-related *Lyssavirus* species 1.

**Fig 3:**
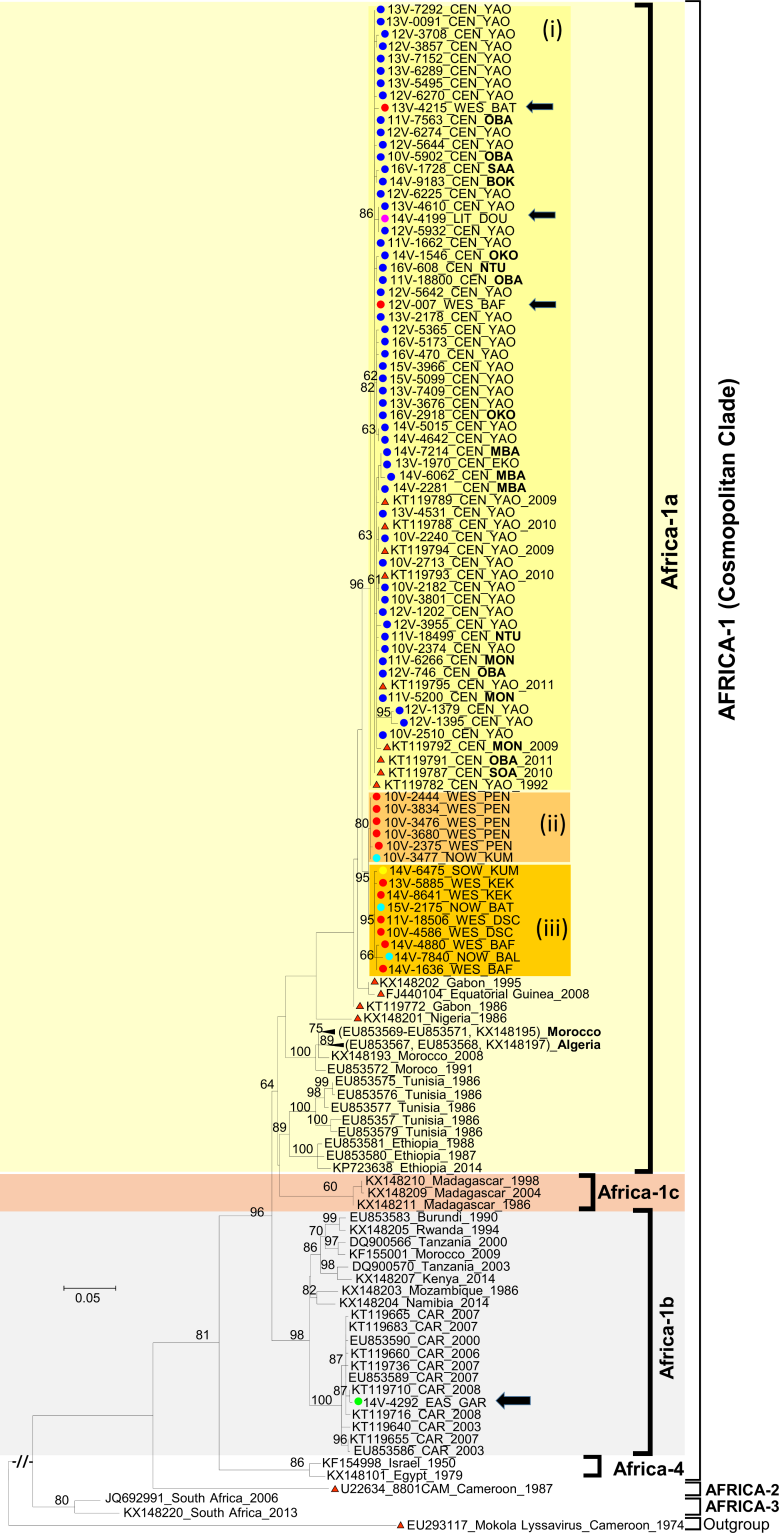
Maximum-likelihood phylogenetic tree of nucleocapsid gene sequences depicting the phylogenetic relationships of Africa-1 Rabies Viruses originating from Cameroon with other Africa-1 from Africa. The phylogenetic tree was estimated from the alignment of 1040 nucleotides (nt) long sequence alignment (positions 41–1080 nucleotides according to the genome of the Rabies Virus strain RRV_ON-99-2) using a maximum-likelihood (ML) method under the general time-reversible (GTR) model of nucleotide substitution, with the rate of each substitution type estimated from the dataset using PHYML 3.0 [55]. The ML base frequencies, the proportion of invariable sites (I) and a gamma distribution of rate variation among sites (Γ with four rate categories) were estimated from the dataset. Newly sequenced Rabies Viruses are indicated with circles color-coded according to their respective regions of origin: CEN, Centre; EAS, East; NOW, North West; SOW, South West and WES, West. Their districts of origin are coded as specified by district codes provided in Table 1. The districts of the Centre region, other than Yaounde, are specified in bold black. The years of origin of the studied viruses are provided by two digit numbers preceding the letter “V” in the virus name (10V-, 2010; 11V-, 2011; 12V-, 2012; 13V-, 2013; 14V-, 2014; 15V-, 2015, 16V-; 2016). Database available reference viruses are named with corresponding GenBank accession numbers followed by the country (CAR, Central African Republic) and year of origin, if known. Viruses from Central Africa are specifically highlighted with red-filed triangles; except those from Cameroon that are further distinguished by the indication of the regions (CEN, Centre) and district (OBA, Obala; MON, Monatélé; SAA, Sa’a; YAO, Yaounde) codes. Clades, subclades and lineages are designated as reported in a reference study based on the ML phylogeny of 321 RABV sequences from five concatenated genes [21]. The major clades, lineages and variants of the Rabies Virus commented in the main text are gathered in color-shaded boxes. Viruses displaying peculiar features, commented in the main text, are further highlighted by black arrows. ML bootstrap values (generated from 100 replicates) >60% are shown next to the nodes. Scale is shown at the left as substitutions per site.

Within the divergent clade of Cosmopolitan RABV, all 71 studied isolates fell in the Africa-1 subclade (Fig 3). None of them grouped with the recently reported Africa-4 subclade defined by RABV isolates from Egypt (in North Africa) and Israel (in Middle East) [34]. Within the Africa-1 subclade, the studied isolates segregated into two distinct lineages with a remarkable association to the geographic origin. One isolate (14V-4292) detected in 2014 in Garoua Boulai (East region) fell within the Africa-1b lineage with previously described isolates from Central African Republic (Fig 3). This isolate displayed 99.9% nt and 100.0% aa sequence identities with its closest match (GenBank N° KT119710) detected in 2008 in the Western part of Central African Republic. Identification of Africa-1b lineage in the East region of Cameroon is consistent with its high prevalence in eastern Africa and in Central African Republic [21,41,44]. This indicates the potential role of intercountry circulation in the sustainment of the enzootic cycle of rabies in Africa.

The other 70 studied isolates of the Africa-1 subclade grouped in the Africa-1a lineage along with previously described isolates from diverse geographic origin in North Africa (Algeria, Ethiopia, Nigeria, and Morocco) and Central Africa (Cameroon, Gabon and Equatorial Guinea) (Fig 3). Within the Africa-1a lineage, studied isolates fell into three distinguishable groups. The first group, (i), was defined by all viruses originating from Yaounde and other districts of the Centre region, indicating some association between the phylogenetic pattern and the regional origin of the RABV isolates from Cameroon. This first reliable group (bootstrap value of 96%) included sequences of RABV previously documented in the Centre region of Cameroon from 1992 to 2010 (Fig 3). However, the apparent feature of in-country geographic differentiation of the RABV was contradicted by several isolates: 12V-007 and 13V-4215, originating respectively from Bafoussam and Batcham in the West region; and 14V-4199 from Douala in the Littoral region. Indeed, they were strikingly related (99.8 to 100% nt and 100.0% aa identity) to their counterparts originating from the Centre region (Fig 3). The second distinguishable group, (ii), gathered Africa-1a isolates originating from the West and North West regions while third group (iii) was defined by Africa-1a isolates from the West, South West and North West regions (Fig 3). These results indicate that RABV circulate in close proximity between geographically close regions.

**Fig 4:**
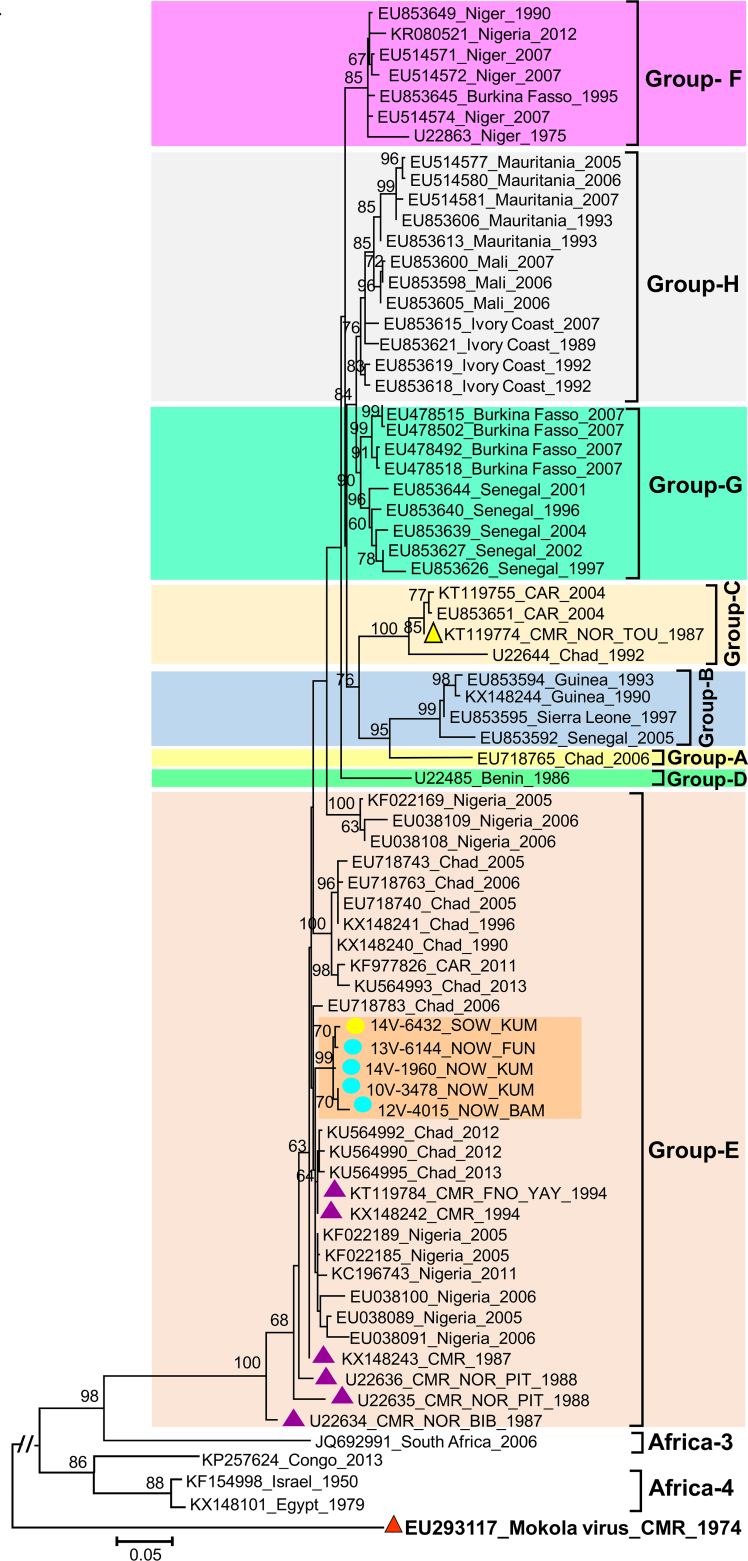
Maximum-likelihood phylogenetic tree of nucleocapsid gene sequences depicting the phylogenetic relationships of Africa-2 Rabies Viruses originating from Cameroon with other Africa-2 from Africa. The phylogenetic tree was estimated from the alignment of 1040 nucleotides (nt) long sequence alignment (positions 41–1080 nucleotides according to the genome of the Rabies Virus strain RRV_ON-99-2) using a maximum-likelihood (ML) method under the K80 model of nucleotide substitution, with the rate of each substitution type estimated from the dataset using PHYML 3.0 [55]. The ML base frequencies, the proportion of invariable sites (I) and a gamma distribution of rate variation among sites (Γ with four rate categories), were estimated from the dataset. Newly sequenced Rabies Virus isolates are indicated with circles color-coded according to their respective regions of origin (NOW, North West and SOW, South West). Their districts of origin, in the North West (BAM; Bamenda; FUN, Fundong; KUM; Kumbo) and South West (KUM, Kumba), are also specified. The years of origin of the studied isolates are provided by two digit numbers preceding the letter “V” in the isolate names (10V-, 2010; 12V-, 2012; 13V-, 2013; 14V-, 2014). Database available reference viruses are named with corresponding GenBank accession numbers followed by the country (CMR, Cameroon; CAR, Central African Republic) and year of origin if known. Those from Cameroon are specifically highlighted with color-coded triangles as follows: yellow for Africa-2 group-C, purple for Africa-2 group-E and red for Mokola virus). Reference viruses from Cameroon are further distinguished by the specification of their respective districts of origin in the North region [(NOR, North): BIB, Bibemy; PIT, Pitoa and TOU, Touboro) and Far North region [(FNO, Far North): YAY, Yaya] if known. Clades, subclades and lineages are designated as reported in a reference study based on the Maximum clade credibility (MCC) of 134 RABV sequences from the nucleocapsid coding genes [36]. The major clades, lineages and variants of the Rabies Virus commented in the main text are gathered in color-shaded boxes. ML bootstrap values (generated from 100 replicates) >60% are shown next to nodes. Scale is shown at bottom as substitutions per site.

Interestingly all five newly sequenced Africa-2 RABV belonged specifically to the group-E (Fig 4). This Africa-2 group-E also comprised RABV strains previously reported in Cameroon and neighboring countries including Central African Republic, Chad, Niger and Nigeria [36,37]. Within the Africa-2 group-E, the newly described isolates were closely related to each other but were loosely related to databases available isolates, including those originating from the Northern regions of Cameroon from 1987 to 1994 (Fig 4). This study revealed that Africa-2 isolates circulate in two southern regions of Cameroon whereas the most prevalent Africa-1 lineage have been documented in five of the seven southern regions.

## Discussion

This study confirms previous reports suggesting continuous circulation of the RABV in Cameroon; especially in the capital city, Yaounde [7,8,54]. In the absence of a multiannual national surveillance and control plan, data on the actual burden of human and animal rabies in Cameroon is certainly underestimated as previously reported in comparable settings in Africa [3,9–12]. This fundamental gap prevents substantial conclusion about the geographical and temporal variation of rabies incidence in Cameroon (Figs 1 and 2).

This study is the first to provide data from elaborate phylogenetic analysis of RABV from Cameroon. Three clades of RABV have been previously documented as being specific to Africa: Cosmopolitan (Africa-1 and Africa-4 subclades), Africa-2 and Africa-3 clades [56]. In this study no isolate of the Africa-3 and Africa-4 was identified. This finding is consistent with the facts that Africa-3 and Africa-4 variants have been shown to be specific to southern [31,51,52] and northern Africa [34], respectively.

Based on the few previous molecular data, it was hypothesized that Africa-2 was exclusively found in the northern part of Cameroon whereas Africa-1 isolates were reported only in the southern regions. This study uncovered RABV of the Africa-2 group-E lineage in two southern regions of Cameroon (North West and South West regions) (Figs 2 and 4). Surprisingly, Africa-2 was the less prevalent lineage in this study whereas it has been shown to be uninterruptedly widespread in western and central Africa, including neighboring countries of Cameroon (Niger, Nigeria, Chad and Central African Republic) [35–37,42,44,45].

This study revealed Africa-1 of the Cosmopolitan clade as the most prevalent RABV lineage circulating in the southern regions of Cameroon (Table 1 and Fig 3). This observation is substantially true for the Centre region of Cameroon where all 51 RABV isolates that could be identified were assigned as Africa-1a lineage. Concerning specifically the North West and South West regions, which share borders with Nigeria, it might be possible to find that Africa-1 and Africa-2 co-circulate with comparable rates if more RABV isolates from these regions were characterized. The Africa-1 sub-clade of the RABV is predominant in the northern, eastern and southern parts of Africa [39,40,57], and have been shown to co-circulate with Africa-2 in Central African Republic [44,45,58] and Nigeria [35,36]. Interestingly, this study provides substantial evidence of co-circulation of Africa-1 and Africa-2 isolates of RABV in at least two regions of the southern part of Cameroon.

In accordance with previous reports, individual RABV sequences fell into a variety of groups, in association with the geographic origin. There was an apparent in-country geographic differentiation of the RABV, however few odds were observed. Similar findings suggesting region-specific variants of the RABV have been documented in some African countries [40,44], thus confirming genomic sequence relatedness as useful marker of intra- and inter-countries RABV dissemination among domestic dogs’ populations. An outstanding application of that marker is provided by the recent finding by Bourhy *et al*., suggesting that the maintenance of the enzootic cycle of rabies at local geographic level in Bangui is more likely driven by human-mediated waves of spread rather than by continuous dispersion in a relatively large and homogenous dogs’ population [45].

A limitation to this study was the fact that no specimen originated from the three northern regions of Cameroon (Fig 2). Furthermore, restricted geographic range covered by the study was associated to the fact that only few specimens originated from the East, Littoral and South regions while as high as 68.0% (68/100) of all specimens were from Yaounde and its neighborhoods (Fig 2). These shortcomings prevent final conclusion to be drawn on the relative rates and genetic diversity of RABV variants co-circulating in Cameroon. In particular, it remains unknown whether the apparently most prevalent Africa-1 lineage of the Cosmopolitan clade circulates in the northern regions of Cameroon (Adamoua, North and Far North regions) (Fig 2). However, our findings suggest that the Cosmopolitan subclade, Africa-1, circulate extensively in the Centre region of Cameroon, and in Yaounde in particular (Fig 2). Meanwhile the Africa-1a lineage was remarkably more frequent, Africa-1b was represented by only one isolate (Fig 3). Given the relatively high rate of Africa-1b RABV reported in the neighboring Central African Republic [44,45,58], it could be hypothesized that the unique Africa-1b identified in this study was introduced from Central African Republic. Accordingly, it has been recently suggested that RABV of the Africa-1 and Africa-2 variants circulate along the trunk roads between Cameroon and the city of Bangui. However, it is not possible to rule out the direction of RABV dissemination across the border between Cameroon and Central African Republic without extensive sampling in both sides.

In contract to the unique Africa-1b isolate, that was strikingly related to their counterparts from Central Africa, Africa-2 viruses were reliably separate from Africa-2 group C previously found along the trunk roads linking the Bangui city to Cameroon [44]. Africa-2 isolates from this study fell within Africa-2 group E and were only loosely related to their counterparts previously reported in the northern part of Cameroon as well as in neighboring countries (Figs 2 and 4). No Africa-2 group-C was found in this study despite the fact that this lineage has been reported in the northern part of Cameroon; that was not covered by this study.

Although it is tempting to explain away failure to efficiently amplify some RABV isolates by low RABV load in 17 studied rabies-positive specimens, the presence of potentially divergent Lyssaviruses among domestic dogs cannot be wiped out. Remarkably, none of the studied isolates displayed close phylogenetic relationships either with the divergent shrew-derived Mokola Lyssavirus strain 86100CAM (GenBank N° EU293117) from Cameroon, or with the Africa-4 and Africa-3 clades which are specific to northern [34] and southern [32,43,52] Africa, respectively. One explanation for the failure to efficiently amplify potential divergent variants of the RABV in this study may be that they were so divergent from the more prevalent variants that they could be refractory to amplification with generic primers used in this study. More powerful experimental approaches (including the use of divergent primers’ systems, virus propagation in cell cultures or high throughput sequencing) will be helpful for the complete assessment of the genetic landscape of *Lyssaviruses* in the studied specimens’ collection. Whole genome sequencing will also help to differentiate genetically-related isolates; thus providing more insights into the spatial dynamics of RABV epidemics.

This study is the first to tackle the molecular epidemiology of RABV isolates in Cameroon. We uncovered the presence of diverse lineages and variants of RABV co-circulating among dog populations in Cameroon. Striking phylogenetic evidence of outcasts to the apparent in-country geographic differentiation of RABV variants provided further support to the idea that the movements of rabid animals may be involved in the spread of dog rabies at least in urban areas. Molecular data reported here constitute potential baseline that would be interesting for the design, optimization and evaluation of rabies surveillance, prevention and control during the future stages of rabies elimination in Central Africa.

## Methods

### Ethical considerations

Animal specimens were collected with the approval of the Cameroon Ministry of Livestock, Fisheries and Animal Industries within the framework of routine rabies surveillance in Cameroon. All studied brain specimens originated from naturally infected rabid dogs enrolled by the Cameroon’s government veterinary services. No specimens were obtained from an experimental procedure nor animals used for experimental purposes.

### Study area

The Republic of Cameroon is a Central African country (Fig 2); sharing borders towards the east with Nigeria, towards the west with Central African Republic, towards the north with Chad and Niger, and towards the south with Equatorial Guinea, Gabon and Congo. Cameroon is characterized by diverse ecosystems that are correlated, and thus attributed to, the patterns of rainfall and geological topology. The highlands of the West and North West regions define an area rich in volcanic lands having an average altitude of ≥ 1,100 meters. The southern rain forest of Cameroon is located in the maritime and equatorial zones (Center, East, Littoral, South and South-West regions) while its northern regions (Adamaoua, North and Far North) are progressively dominated by the savannah and steppe. There is no geographical features that may represent barriers to rabies spread in Cameroon. According to the 2010 estimates, the population of the Cameroon is at 19,406,100 people with 10,091,172 and 9,314,928 inhabitants in urban and rural settings, respectively [59]. In particular, the Center region has approximately 2,638,648 inhabitants in urban settings as compared to 887,016 people in rural settings.

### Specimens

This was a retrospective and transversal study based on the biological collection of brain specimens collected from rabid domestic dogs. Originally, heads of domestic dogs suspected of rabies were obtained from both private and public veterinary services. Domestic dogs were suspected of rabies if they displayed at least two of the following signs and symptoms: unprovoked aggression, foaming at the mouth, paralysis, incoordination, hoarse bark, hydrophobia, weakness, seizures, or loss of appetite. Brain specimens were collected during necropsy performed on dogs’ heads submitted for rabies diagnosis at the Centre Pasteur du Cameroun (CPC) located in the capital city, Yaounde. Laboratory confirmation of rabies at CPC was based on the detection of RABV nucleocapsid antigen in brain specimens by direct Fluorescent Antibody Test (dFAT) using rabbit IgG antibodies (Bio-Rad, Marnes-la-Coquette, France) [8]. Brain specimens negative for FAT were further confirmed by virus isolation on Murina neuroblastoma cell cultures as previously described [60]. After rabies diagnosis, remaining brain samples were kept frozen at -80°C. A total of 100 specimens derived from 100 rabid dogs were available and were thus considered in this study. All studied specimens originated from the 7 southern regions of Cameroon from 2010 to 2016 (Table 1 and Fig 2).

### RNA extraction and amplification of RABV nucleocapsid gene

Each brain specimen was crushed in PBS (10% weight/volume) and 250 μL of supernatant resulting from clarified brain suspension was subjected to RNA extraction using TRIzol LS (Invitrogen, Paris, France), as recommended by the manufacturer's instructions. RNA samples were stored at -80°C prior to analysis.

Complementary DNA (cDNA) synthesis was performed in a final volume of 20 μL using pd(N)_7_ random primers and AMV reverse transcriptase (Promega). Briefly, 7 μL of purified RNA was incubated at 65°C for 10 minutes with 2 μL of RNase- and DNase-free water, 100 ng of random primers (1 μL) and 10 nmol. of each deoxynucleotide triphosphate (1 μL). Reaction tubes were then transferred on ice for at least 2 minutes and completed with 3 μL of RNase- and DNase-free water, 4 μL of AMV 5X Buffer, 40 U of RNAsin (1 μL) and 10 U of AMV reverse transcriptase (1 μL). Resulting 20 μL reaction mixtures were incubated 10 min at 25°C, 90 min at 42°C and 5 min at 95°C.

A 1485-base pairs DNA fragment encompassing the entire 1353 nucleotides (nt) of the N gene of RABV was amplified by reverse transcription-nested polymerase chain reaction (RT-nPCR) using previously described consensus oligonucleotide primers. First round PCR was performed with the primers pair RHN1 (5’-ACAGACAGCGTCAATTGCAAAGC-3’, nucleotides (nt) 28–52) and N8m (5’-CAGTCTCYTCNGCCATCTC-3’; nt 1584–1568) [21,26,61] in a final volume of 50 μL containing: 5 μL of cDNA, 34.5 μL of RNase- and DNase-free water, 5 μL of 10X PCR Buffer, 200 μM of each dNTP, 2 mM of MgCl_2_, 25 pmol of each primers and 2.5U of Taq DNA polymerase (Invitrogen, Cergy-Pontoise, France). The thermocycler profile was as follows: 5 min at 95°C followed by 35 cycles of 30 s at 95°C, 30 s at 56°C and 2 min at 72°C, and a final elongation at 72°C for 10 min. Second round PCR was carried out from 2 μL of the PCR product using the primers pair N127 (5’-ATGTAACACCTCTACAATGG-3’, nt 55–74) and 304 (5’-GAGTCACTCGAATATGTC-3’; nt 1539–1516) [21,62] under the same experimental conditions.

PCR products were analyzed by migration on Gelgreen-stained agarose gels and reveled on an ultraviolet transilluminator.

### Sequencing and sequences analyses

Amplicons were purified using the QIAquick PCR Purification kit (Qiagen, Courtaboeuf, France) following the manufacturer’s protocol. Purified amplicons were subjected to direct double strands sequencing using nested PCR primers, the BigDye terminator v3.1 kit (Applied Biosystems) and the ABI Prism 3140 automated sequencer (Applied Biosystems).

Consensus sequence editing, multiple sequences alignments and pairwise sequence comparisons were carried out with the CLC Main Workbench 5.7.2 software (CLC bio, Aarhus, Denmark).

GenBank accession numbers for the nucleocapsid gene sequences of RABV determined in this study have been assigned as MF537505 to MF537580.

### Phylogenetic analyses

To determine the phylogenetic relatedness of the newly sequenced RABV isolates, sequences were originally aligned with all relevant reference sequences available from online databases and originating from Cameroon and neighboring countries as well as representative sequences from other parts of Africa. Based on initial trees obtained, the alignment was downsized by removing duplicate sequences and by splitting the alignment into two datasets. We used Smart Model Selection [63] to determine the best-fit model of nucleotide substitution based on the Bayesian Information Criterion. This revealed that the General Time Reversible model with proportion of invariable sites plus gamma-distributed rate heterogeneity (GTR+I+Γ4) was optimal for the Africa 1 related dataset while the K80 model with proportion of invariable sites plus gamma-distributed rate heterogeneity (K80+I+Γ4) was the most suitable for the Africa 2 related dataset. Phylogenetic trees using individual datasets were then estimated by the maximum likelihood (ML) method available in PhyML 3.0 [55] using SPR branch-swapping. The reliability of individual nodes on the phylogenetic trees was estimated using 1,000 bootstrap pseudoreplicates.

## Supporting information

S1 Table List of reference Rabies Viruses whose nucleocapsid coding gene sequences were used in the analyses. (PDF)
