# Supplementary material for: Molecular characterization and phylogenetic relatedness of dog-derived Rabies Viruses circulating in Cameroon between 2010 and 2016
Source: PLoS Negl Trop Dis. 2017 Oct 30;11(10):e0006041. doi: 10.1371/journal.pntd.0006041 (PMC5679643; doi:10.1371/journal.pntd.0006041)
Supplement: S1 Table — (PDF) [file pntd.0006041.s001.pdf]

Table S1. List of reference Rabies Viruses whose nucleocapsid coding gene sequences were used in the analyses.

Country	Virus Name	Host (Scientific name)[a]	Year	Phylogenetic clade (subclade)[b]	Accession number	Reference
Algeria	15006FRA	Dog (*Canis familiaris*)	2015	Cosmopolitan (AF-1a)	KX148197	[1]
Algeria	96018ALG	Human (*Homo sapiens*)	1996	Cosmopolitan (AF-1a)	EU853567	[2]
Algeria	96019ALG	Human (*Homo sapiens*)	1996	Cosmopolitan (AF-1a)	EU853568	[2]
Benin	8697BEN	Cat *(Felis catus)*	1986	Africa-2	U22485	[3]
Burkina Faso	139BF	Dog (*Canis familiaris*)	2007	Africa-2	EU478515	[2]
Burkina Faso	36BF	Dog (*Canis familiaris*)	2007	Africa-2	EU478502	[2]

Burkina Faso	28BF	Dog (*Canis familiaris*)	2007	Africa-2	EU478492	[2]
Burkina Faso	49BF	Dog (*Canis familiaris*)	2007	Africa-2	EU478518	[2]
Burkina Faso	9547HAV	Dog (*Canis familiaris*)	1995	Africa-2	EU853645	[2]
Cameroon	9236CAM	Dog (*Canis familiaris*)	1992	Cosmopolitan (AF-1a)	KT119782	[4]
Cameroon	11018CAM	Dog (*Canis familiaris*)	2009	Cosmopolitan (AF-1a)	KT119787	[4]
Cameroon	11019CAM	Dog (*Canis familiaris*)	2010	Cosmopolitan (AF-1a)	KT119788	[4]
Cameroon	11020CAM	Dog (*Canis familiaris*)	2009	Cosmopolitan (AF-1a)	KT119789	[4]
Cameroon	11027CAM	Dog (*Canis familiaris*)	2010	Cosmopolitan (AF-1a)	KT119793	[4]
Cameroon	11023CAM	Dog (*Canis familiaris*)	2011	Cosmopolitan (AF-1a)	KT119791	[4]
Cameroon	11025CAM	Dog (*Canis familiaris*)	2009	Cosmopolitan (AF-1a)	KT119792	[4]

Cameroon	11028CAM	Dog (*Canis familiaris*)	2011	Cosmopolitan (AF-1a)	KT119794	[4]
Cameroon	11029CAM	Dog (*Canis familiaris*)	2011	Cosmopolitan (AF-1a)	KT119795	[4]
Cameroon	88003CAM	Dog (*Canis familiaris*)	1987	Africa-2	KX148243	[1]
Cameroon	95002CAM	Dog (*Canis familiaris*)	1994	Africa-2	KX148242	[1]
Cameroon	9502CAM	Dog (*Canis familiaris*)	1994	Africa-2	KT119784	[4]
Cameroon	8802CAM	Dog (*Canis familiaris*)	1987	Africa-2	KT119774	[4]
Cameroon	8801CAM	Dog (*Canis familiaris*)	1987	Africa-2	U22634	[3]
Cameroon	8804CAM	Dog (*Canis familiaris*)	1988	Africa-2	U22635	[2]
Cameroon	8805CAM	Dog (*Canis familiaris*)	1988	Africa-2	U22636	[2]

Cameroon	86100CAM	Shrew	1974	Mokola Lyssavirus	EU293117	[5]
Central African Republic	08275RCA	Dog (*Canis familiaris*)	2008	Cosmopolitan (AF-1b)	KT119710	[4]
Central African Republic	09041RCA	Dog (*Canis familiaris*)	2009	Cosmopolitan (AF-1b)	KT119710	[4]
Central African Republic	09041RCA	Dog (*Canis familiaris*)	2009	Cosmopolitan (AF-1b)	KT119736	[4]
Central African Republic	07134RCA	Dog (*Canis familiaris*)	2003	Cosmopolitan (AF-1b)	KT119640	[4]
Central African Republic	07150RCA	Dog (*Canis familiaris*)	2004	Cosmopolitan (AF-1b)	KT119655	[4]
Central African Republic	07157RCA	Dog (*Canis familiaris*)	2006	Cosmopolitan (AF-1b)	KT119660	[4]
Central African Republic	07160RCA	Dog (*Canis familiaris*)	2007	Cosmopolitan (AF-1b)	KT119665	[4]
Central African Republic	7180RCA	Dog (*Canis familiaris*)	2007	Cosmopolitan (AF-1b)	KT119683	[4]

Central African Republic	07128RCA	Dog (*Canis familiaris*)	2003	Cosmopolitan (AF-1b)	EU853586	[2]
Central African Republic	7189RCA	Dog (*Canis familiaris*)	2007	Cosmopolitan (AF-1b)	EU853589	[2]
Central African Republic	07072RCA	Dog (*Canis familiaris*)	2000	Cosmopolitan (AF-1b)	EU853590	[2]
Central African Republic	CAR_11_001	Human (*Homo sapiens*)	2011	Africa-2	KF977826	[6]
Central African Republic	othercity_07151RCA_2004_708	Dog (*Canis familiaris*)	2004	Africa-2	KT119755	[4]
Central African Republic	07149RCA	Dog (*Canis familiaris*)	2004	Africa-2	EU853651	[2]
Chad	90021TCH	Dog (*Canis familiaris*)	1990	Africa-2	KX148240	[1]
Chad	96009TCH	Dog (*Canis familiaris*)	1996	Africa-2	KX148241	[1]
Chad	9218TCH	Dog (*Canis familiaris*)	1992	Africa-2	U22644	[3]

Chad	369	Dog (*Canis familiaris*)	2013	Africa-2	KU564995	Lepelletier *et al.* (unpublished)
Chad	359	Dog (*Canis familiaris*)	2012	Africa-2	KU564990	Lepelletier *et al.* (unpublished)
Chad	364	Dog (*Canis familiaris*)	2012	Africa-2	KU564992	Lepelletier *et al.* (unpublished)
Chad	367	Dog (*Canis familiaris*)	2013	Africa-2	KU564993	Lepelletier *et al.* (unpublished)
Chad	Chad2006_45	Dog (*Canis familiaris*)	2006	Africa-2	EU718783	[2]
Chad	Chad_36_193	Dog (*Canis familiaris*)	2005	Africa-2	EU718740	[2]
Chad	Chad_31_188	Dog (*Canis familiaris*)	2005	Africa-2	EU718743	[7]
Chad	Chad_42_199	Dog (*Canis familiaris*)	2006	Africa-2	EU718763	[2]
Egypt	86092EGY	Human (*Homo sapiens*)	1979	Cosmopolitan (AF-4)	KX148101	[1]

Equatorial Guinea	eg1	Human (*Homo sapiens*)	2008	Cosmopolitan (AF-1a)	FJ440104	[8]
Ethiopia	9531ETH	Cow	1987	Cosmopolitan (AF-1a)	EU853580	[2]
Ethiopia	8808ETH	Dog (*Canis familiaris*)	1988	Cosmopolitan (AF-1a)	EU853581	[2]
Ethiopia	RV2985	Ethiopian wolf (*Canis simensis*)	2014	Cosmopolitan (AF-1a)	KP723638	[9]
Gabon	8698GAB	Dog (*Canis familiaris*)	1986	Cosmopolitan (AF-1a)	KT119772	[4]
Gabon	95049GAB	Dog (*Canis familiaris*)	1995	Cosmopolitan (AF-1a)	KX148202	[1]
Guinea	90024GUI	Dog (*Canis familiaris*)	1990	Africa-2	KX148244	[1]
Guinea	9361GUI	Dog (*Canis familiaris*)	1993	Africa-2	EU853594	[2]
Israel	RV2324	Dog (*Canis familiaris*)	1950	Cosmopolitan (AF-4)	KF154998	[9]
Ivory Coast	07059IC	Dog (*Canis familiaris*)	2007	Africa-2	EU853615	[2]
Ivory Coast	9237IC	Dog (*Canis familiaris*)	1992	Africa-2	EU853618	[2]

Ivory Coast	9238IC	Dog (*Canis familiaris*)	1992	Africa-2	EU853619	[2]
Ivory Coast	8913IC	Dog (*Canis familiaris*)	1989	Africa-2	EU853621	[2]
Kenya	14015ITA	Human (*Homo sapiens*)	2014	Cosmopolitan (AF-1b)	KX148207	[1]
Mali	07232MAL	Dog (*Canis familiaris*)	2006	Africa-2	EU853605	[2]
Mali	07235MAL	Dog (*Canis familiaris*)	2006	Africa-2	EU853598	[2]
Mali	07217MAL	Dog (*Canis familiaris*)	2007	Africa-2	EU853600	[2]
Madagascar	04033MAD	Dog (*Canis familiaris*)	2004	Cosmopolitan (AF-1c)	KX148209	[1]
Madagascar	86046MAD	Dog (*Canis familiaris*)	1986	Cosmopolitan (AF-1c)	KX148211	[1]
Madagascar	98002MAD	Human (*Homo sapiens*)	1998	Cosmopolitan (AF-1c)	KX148210	[1]

Mauritania	9311MAU	Dog (*Canis familiaris*)	1993	Africa-2	EU853606	[2]
Mauritania	9133MAU	Ass	1991	Africa-2	EU853613	[2]
Mauritania	1923MAU/05	Dog (*Canis familiaris*)	2005	Africa-2	EU514577	[2]
Mauritania	2019MAU/06	Dog (*Canis familiaris*)	2006	Africa-2	EU514580	[2]
Mauritania	2049MAU/07	Goat	2007	Africa-2	EU514581	[2]
Mozambique	86031MOZ	Dog (*Canis familiaris*)	1986	Cosmopolitan (AF-1b)	KX148203	[1]
Morocco	9016MAR	Dog (*Canis familiaris*)	1990	Cosmopolitan (AF-1a)	EU853569	[2]
Morocco	9109MAR	Human (*Homo sapiens*)	1991	Cosmopolitan (AF-1a)	EU853570	[2]
Morocco	9108MAR	Human (*Homo sapiens*)	1991	Cosmopolitan (AF-1a)	EU853571	[2]
Morocco	8678MAR	Human (*Homo sapiens*)	1986	Cosmopolitan (AF-1a)	EU853572	[2]

Morocco	04031MAR	Dog (*Canis familiaris*)	2004	Cosmopolitan (AF-1a)	KX148195	[1]
Morocco	08342MAR	Dog (*Canis familiaris*)	2008	Cosmopolitan (AF-1a)	KX148193	[1]
Morocco	RV2627	Cow (*Bos taurus*)	2009	Cosmopolitan (AF-1b)	KF155001	[9]
Namibia	92030NAM	Dog (*Canis familiaris*)	1992	Cosmopolitan (AF-1b)	KX148204	[1]
Niger	9013NIG	Dog (*Canis familiaris*)	1990	Africa-2	EU853649	[2]
Niger	246NIG/07	Dog (*Canis familiaris*)	2007	Africa-2	EU514571	[2]
Niger	247NIG/07	Dog (*Canis familiaris*)	2007	Africa-2	EU514572	[2]
Niger	252NIG/07	Dog (*Canis familiaris*)	2007	Africa-2	EU514574	[2]
Niger	8718NIG	Dog (*Canis familiaris*)	1975	Africa-2	U22863	[3]
Nigeria	86003BRE	Dog (*Canis familiaris*)	1986	Cosmopolitan (AF-1a)	KX148201	[1]

Nigeria	RD128	Dog (*Canis familiaris*)	2006	Africa-2	EU038108	[2]
Nigeria	RD141HN	Dog (*Canis familiaris*)	2006	Africa-2	EU038109	[2]
Nigeria	D7305HN	Dog (*Canis familiaris*)	2005	Africa-2	EU038089	Dzikwi,A.A *et al*., (unpublished)
Nigeria	RD41HN	Dog (*Canis familiaris*)	2006	Africa-2	EU038091	[2]
Nigeria	RD103	Dog (*Canis familiaris*)	2006	Africa-2	EU038100	[2]
Nigeria	9N/2005	Dog (*Canis familiaris*)	2005	Africa-2	KF022169	David D. (unpublished)
Nigeria	155N/2005	Dog (*Canis familiaris*)	2005	Africa-2	KF022185	David D. (unpublished)
Nigeria	177N/2005	Dog (*Canis familiaris*)	2005	Africa-2	KF022189	David D. (unpublished)
Nigeria	DRV-NG11	Dog (*Canis familiaris*)	2011	Africa-2	KC196743	[10]

Nigeria	252NigeriaMinna2012	Dog (*Canis familiaris*)	2012	Africa-2	KR080521	Velasco-Villa,A. *et al.*, (unpublished)
Rwanda	94289RWA	Dog (*Canis familiaris*)	1994	Cosmopolitan (AF1-b)	KX148205	[1]
Senegal	07207SEN	Human (*Homo sapiens*)	1997	Africa-2	EU853626	[2]
Senegal	07020SEN	Human (*Homo sapiens*)	2002	Africa-2	EU853627	[2]
Senegal	07021SEN	Human (*Homo sapiens*)	2004	Africa-2	EU853639	[2]
Senegal	07019SEN	Human (*Homo sapiens*)	1996	Africa-2	EU853640	[2]
Senegal	02025SEN	Human (*Homo sapiens*)	2001	Africa-2	EU853644	[2]
Senegal	07023SEN	Human (*Homo sapiens*)	2005	Africa-2	EU853592	[2]
Sierra Leone	07208SL	cat	1997	Africa-2	EU853595	[2]
South Africa	15001AFS	Yellow mongoose (*Cynictis penicillata)*	2013	Africa-3	KX148220	[1]

South Africa	956-06	Mongoose	2014	Africa-3	JQ692991	Ngoepe E. *et al.,* (unpublished)
Tanzania	RV853	spotted hyena	2000	Cosmopolitan (AF-1b)	DQ900566	[11]
Tanzania	A04-4981	small-spotted genet	2003	Cosmopolitan (AF-1b)	DQ900566	[11]

[a] The scientific name of host species is provided, if available.
[b] AF-1a, lineage "a" of the subclade Africa-1; AF-1b, lineage "b" of the subclade Africa-1, AF-1c, lineage "c" of the subclade Africa-1.
ND, no data available.

REFERENCES

1. Troupin C, Dacheux L, Tanguy M, Sabeta C, Blanc H, et al. (2016) Large-Scale Phylogenomic Analysis Reveals the Complex Evolutionary History of Rabies Virus in Multiple Carnivore Hosts. PLoS Pathog 12: e1006041.
2. Talbi C, Holmes EC, de Benedictis P, Faye O, Nakoune E, et al. (2009) Evolutionary history and dynamics of dog rabies virus in western and central Africa. J Gen Virol 90: 783-791.
3. Kissi B, Tordo N, Bourhy H (1995) Genetic polymorphism in the rabies virus nucleoprotein gene. Virology 209: 526-537.
4. Bourhy H, Nakoune E, Hall M, Nouvellet P, Lepelletier A, et al. (2016) Revealing the Micro-scale Signature of Endemic Zoonotic Disease Transmission in an African Urban Setting. PLoS Pathog 12: e1005525.
5. Delmas O, Holmes EC, Talbi C, Larrous F, Dacheux L, et al. (2008) Genomic diversity and evolution of the lyssaviruses. PLoS One 3: e2057.
6. Tricou V, Berthet N, Nakoune E, Kazanji M (2014) Complete genome sequence of a rabies virus isolated from a human in central african republic. Genome Announc 2.
7. Durr S, Naissengar S, Mindekem R, Diguimbye C, Niezgoda M, et al. (2008) Rabies diagnosis for developing countries. PLoS Negl Trop Dis 2: e206.

8. Rubin J, David D, Willoughby RE, Jr., Rupprecht CE, Garcia C, et al. (2009) Applying the Milwaukee protocol to treat canine rabies in Equatorial Guinea. Scand J Infect Dis 41: 372-375.
9. Marston DA, Wise EL, Ellis RJ, McElhinney LM, Banyard AC, et al. (2015) Complete genomic sequence of rabies virus from an ethiopian wolf. Genome Announc 3.
10. Zhou M, Zhou Z, Kia GS, Gnanadurai CW, Leyson CM, et al. (2013) Complete genome sequence of a street rabies virus isolated from a dog in Nigeria. Genome Announc 1.
11. Lembo T, Haydon DT, Velasco-Villa A, Rupprecht CE, Packer C, et al. (2007) Molecular epidemiology identifies only a single rabies virus variant circulating in complex carnivore communities of the Serengeti. Proc Biol Sci 274: 2123-2130.
